# A Novel R2R3–MYB Transcription Factor FaMYB10-like Promotes Light-Induced Anthocyanin Accumulation in Cultivated Strawberry

**DOI:** 10.3390/ijms242316561

**Published:** 2023-11-21

**Authors:** Yiping Wang, Yongqiang Liu, Lianxi Zhang, Li Tang, Shiqiong Xu, Zikun Wang, Yunting Zhang, Yuanxiu Lin, Yan Wang, Mengyao Li, Yong Zhang, Ya Luo, Qing Chen, Haoru Tang

**Affiliations:** College of Horticulture, Sichuan Agricultural University, Chengdu 611130, China; wangyp_sicau@163.com (Y.W.); liuyq0129@163.com (Y.L.); zlx981027@163.com (L.Z.); tangli_sicau@163.com (L.T.); xushiqiong1999@163.com (S.X.); 13980422535@163.com (Z.W.); asyunting@sicau.edu.cn (Y.Z.); linyx@sicau.edu.cn (Y.L.); wangyanwxy@163.com (Y.W.); limy@sicau.edu.cn (M.L.); zhyong@sicau.edu.cn (Y.Z.); luoya945@sicau.edu.cn (Y.L.); supnovel@sicau.edu.cn (Q.C.)

**Keywords:** strawberry, anthocyanin, light, MYB, tissue specific

## Abstract

Anthocyanins widely accumulate in the vegetative and reproductive tissues of strawberries and play an important role in stress resistance and fruit quality. Compared with other fruits, little is known about the molecular mechanisms regulating anthocyanin accumulation in strawberry vegetative tissues. In this study, we revealed an R2R3–MYB transcription factor, FaMYB10-like (FaMYB10L), which positively regulated anthocyanin accumulation and was induced by light in the petiole and runner of cultivated strawberry. FaMYB10L is a homologue of FveMYB10-like and a nuclear localization protein. Transient overexpression of *FaMYB10L* in a white fruit strawberry variety (*myb10* mutant) rescued fruit pigmentation, and further qR–PCR analysis revealed that FaMYB10L upregulated the expression levels of anthocyanin biosynthesis-related genes and transport gene. A dual luciferase assay showed that FaMYB10L could activate the anthocyanin transport gene *FaRAP*. Anthocyanin accumulation was observed in FaMYB10L-overexpressing strawberry calli, and light treatment enhanced anthocyanin accumulation. Furthermore, transcriptomic profiling indicated that the DEGs involved in the flavonoid biosynthesis pathway and induced by light were enriched in *FaMYB10L*-overexpressing strawberry calli. In addition, yeast two-hybrid assays and luciferase complementation assays indicated that FaMYB10L could interact with bHLH3. These findings enriched the light-involved regulatory network of anthocyanin metabolism in cultivated strawberries.

## 1. Introduction

Cultivated strawberries (*Fragaria × ananassa*) are aromatic, juicy, nutritious, and popular among consumers and are cultivated worldwide. Anthocyanins are the principal pigments responsible for the brilliant red colour of strawberry fruits [1]. Anthocyanins play different roles in various tissues. In fruits, anthocyanins have a significant function in improving fruit quality; in flowers, they attract pollinators and dispersers; in vegetative tissues, anthocyanins enhance plant resistance to external stresses; and in seeds, anthocyanins act as endogenous antioxidants to protect the chemical composition and aid in seed dormancy [2,3].

Anthocyanin biosynthesis is a branch of the flavonoid biosynthetic pathways [4]. With further research, the biosynthetic process of anthocyanin in strawberries has been elucidated, especially the isolation and functional identification of key enzyme genes [5]. With phenylalanine as the precursor, it is catalyzed by key enzymes, which are classified as early biosynthesis-related genes (EBGs) and late biosynthesis-related genes (LBGs) and converted into anthocyanins and stored in vacuoles. These biosynthesis-related genes have been reported in strawberries, and their loss of function causes an abnormal colouration [6,7,8,9,10]. Additionally, one of the other crucial steps is the transfer to the vacuole [11]. GSTs likely play the most significant role among the transporters, and they frequently result in significant alterations in fruit coloration. For example, in Arabidopsis, GST (TT19) loss-of-function mutants have been found, such as with abnormal anthocyanin accumulation [12]. In strawberries, RAP is the primary GST transporter of anthocyanin of all the paralogues and mediates fruit pigmentation [13]. This biosynthetic process is regulated by MYB TFs, bHLH TFs, and WD40 proteins, which are named MBW complexes [14,15].

The MYB transcription factor is the core transcription factor in anthocyanin biosynthesis regulation, and its expression has strong tissue specificity [16]. In apples, of the allelic homologues, fruit flesh and foliage anthocyanin accumulation have been demonstrated to be regulated by MdMYB10, whereas *MdMYB1* and *MdMYBA* are expressed in the fruit skin [17,18,19]. Some MYBs have also been characterized in strawberries, including FaMYB10 [20], FaMYB123 [21], and an anthocyanin biosynthesis inhibitor FaMYB1 [22]. Among them, MYB10 codes for a dominant regulatory transcription factor are involved in the anthocyanin accumulation of strawberry fruits. The mature fruits of *FvMYB10* RNAi lines [15], wild strawberry “Yellow Wonder YW5AF7” (*myb10* mutant) [23], and cultivated strawberry “Snow White” (*myb10* mutant) all had no pigmented fruit [24]. Moreover, anthocyanin accumulated in the vegetative tissues of these *myb10*-loss-function mutants indicate the existence of regulators other than MYB10, which influence anthocyanin accumulation in strawberries. Moreover, environmental cues also have an impact on the MYB-mediated control of anthocyanin accumulation at the transcriptional and posttranslational stages [25].

Light is one of the most important environmental factors regulating anthocyanin accumulation [26,27,28]. Light signal transduction factors positively regulate anthocyanin accumulation by activating key MYB regulators [29]. MYB responds to light at both the transcript and protein levels, thereby affecting the accumulation of anthocyanin [30,31]. When exposed to external stresses, such as strong light, the vegetative tissues of cultivated strawberries will accumulate a large amount of anthocyanin to resist the stress. It is significant to analyze the key regulatory factors for improving cultivation measures to improve strawberry quality. Through an analysis of a previous transcriptome of cultivated strawberry calli under light/dark treatment, we obtained a light-responsive R2R3–MYB transcription factor, FaMYB10L, which is a homologue of FveMYB10L [32]. In our study, *FaMYB10L* possessed low expression levels in strawberry fruits, highly expressed in petioles and runners and induced by light. Transient overexpression in the white fruit strawberry variety “Snow White” (*myb10* mutant) rescued pigmentation. *FaMYB10L*-overexpressing strawberry calli showed a light-dependent increase in anthocyanin accumulation. Meanwhile, FaMYB10L could increase the transcript levels of anthocyanin biosynthesis-related genes and transport genes. In summary, we identified a novel transcription factor that regulated anthocyanin accumulation in the vegetative tissues of cultivated strawberries and identified its effect on anthocyanin accumulation through transient and stable genetic transformation. These results may enrich the anthocyanin biosynthesis network regulated by light signals in cultivated strawberries.

## 2. Results

### 2.1. Identification and Sequence Analysis of FaMYB10L

**Figure 1:**
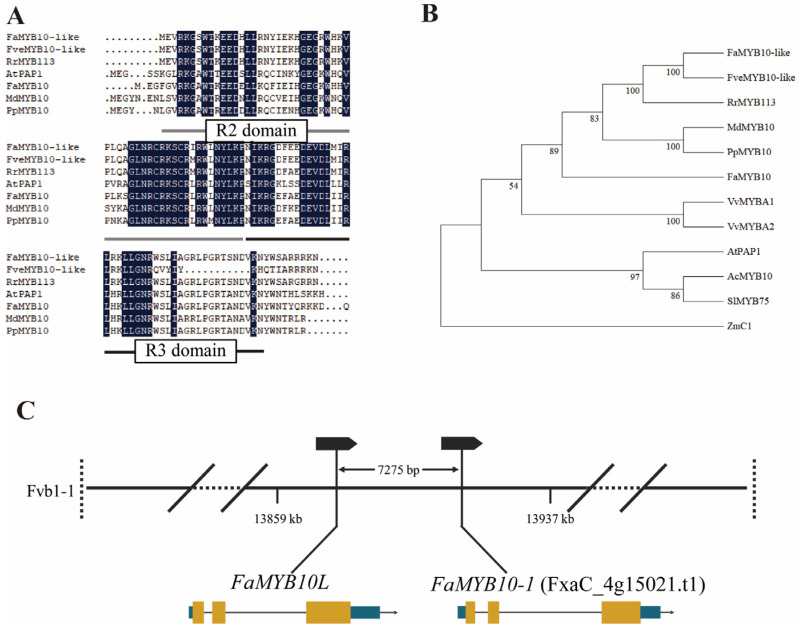
Alignment, phylogenetic relationship, and chromosome location of FaMYB10L. (**A**) Multiple protein sequence alignment. (**B**) Phylogenetic relationships between FaMYB10L and its closest homologues in different species. ClustalW was used to perform multiple sequence alignments and the neighbour-joining method with 1000 bootstrap replications, which was used to build the phylogenetic tree. Accession IDs (GenBank): RrMYB113 (*Rosa rugosa*), AXQ12351.1; MdMYB10 (*Malus domestica*), ACQ45201.1; PpMYB10 (*Prunus persica*), ADK73605.1; VvMYBA1 (*Vitis vinifera*), BAD18977.1; FaMYB10 (*Fragaria x ananassa*), USN17647.1; BAD18978.1; AtPAP1 (*Arabidopsis thaliana*), VvMYBA2 (*Vitis vinifera*), AEE33419.1; AcMYB10 (*Actinidia chinensis*), QGA78460.1; SlMYB75 (*Solanum lycopersicum*), NP_001265992.1; ZmC1 (*Zea mays*), AAK81903. (**C**) Schematic diagram of FaMYB10L and FaMYB10-1 located on chromosome Fvb1–1.

In our previous transcriptome analysis, we identified a light-responsive MYB gene in cultivated strawberries, *FaMYB10L* (Genebank, OR399553) (Supplementary Figure S1), which was the homologue of FveMYB10-like. Amino acid sequence analysis showed that FaMYB10L has complete R2 and R3–MYB domains (Figure 1A), which are typical R2R3–MYB transcription factors. A phylogenetic analysis showed FaMYB10L to be a close homologue of FveMYB10-like, RrMYB113, FaMYB10, MdMYB10, PpMYB10.1, and PavMYB10 in rosaceous plants (Figure 1B), which are related to anthocyanin production. Alignment to the octoploid cv Camarosa genome, *FaMYB10L* is located on chromosome Fvb1–1 [33]. In addition, *FaMYB10L* resides near *FaMYB10–1* (FxaC_4g15021.t1) on Chromosome Fvb1–1 approximately 7000 bp apart (Figure 1C).

### 2.2. Subcellular Localization and Transcriptional Activity Analysis of FaMYB10L

**Figure 2:**
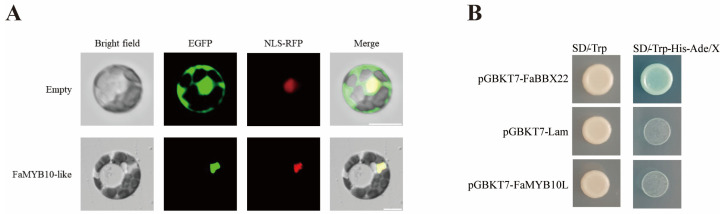
Transcription factor characteristic analysis of FaMYB10L. (**A**) Subcellular localization of the FaMYB10L protein in strawberry protoplasts. NLS–RFP, a nuclear marker; Bars, 10 µm. (**B**) Transcriptional activity analysis of FaMYB10L. pGBKT7–FaBBX22, positive control; pGBKT7–Lam, negative control.

We fused the FaMYB10L CDS with the EGFP-encoding gene and transiently cotransformed the fusion construct and an NLS–RFP (nuclear location marker) construct into strawberry protoplasts. EGFP detection revealed that FaMYB10L is completely confined to the nucleus. In contrast, only EGFP was found throughout the cell. These results demonstrated that FaMYB10L is a nuclear protein (Figure 2A).

To investigate the transcriptional activity of FaMYB10L, a transactivation assay was performed in Yeast Strain Y2HGold. The yeast cells that were transformed with BD–FaMYB10L (pGBKT7–FaMYB10L) could not grow on the SD/–Trp–His–Ade/X–α–gal medium, indicating that FaMYB10L has no transacting ability in yeast cells (Figure 2B).

### 2.3. The Expression Patterns of FaMYB10L

**Figure 3:**
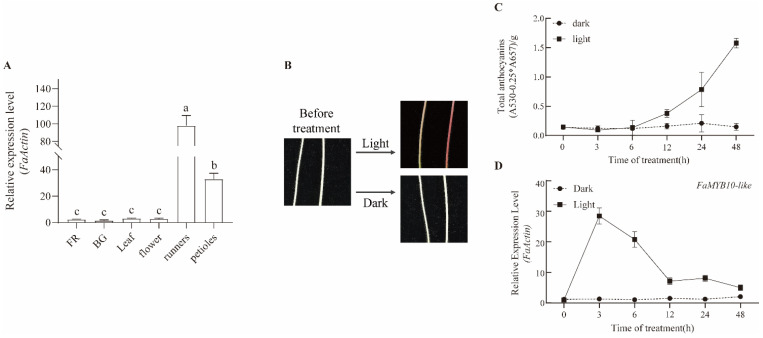
The expression patterns of *FaMYB10L*. (**A**) Tissue-specific expression level of *FaMYB10L*. FR, full red; BG, big green. (**B**) Anthocyanin accumulation in “Benihoppe” strawberry petioles after light treatment. (**C**) Anthocyanin contents of petioles changed with light treatment. (**D**) Relative expression level of *FaMYB10L* during treatment. The error bars for expression level and total anthocyanin contents show the SD of 3 independent replicates. Different letters above the bars indicate significantly different values (*p* < 0.05) according to a Least Significant Difference (LSD) test.

qRT–PCR was used to analyze the spatial and temporal expression patterns of *FaMYB10L*. *FaMYB10L* was lowly or not expressed in strawberry fruits, but it was highly expressed in petioles and runners of cultivated strawberries (Figure 3A).

The influences of light on the transcription level of *FaMYB10L* were evaluated in “Benihoppe” strawberry petioles exposed to continuous light (Figure 3B). Anthocyanin content increased in response to the treatment after 6 h and continued to increase over the period of the treatment (48 h) (Figure 3C). As a result of the light treatment, *FaMYB10L* expression was upregulated within 3 h. (Figure 3D). Furthermore, a promoter sequence ~1.8 kb upstream of the *FaMYB10L* transcriptional start site (Supplementary Figure S2) was fused to the β–glucuronidase reporter gene (*Gus*). The *ProFaMYB10L*::*Gus* construct was transiently transformed into tobacco leafs. qRT–PCR analysis showed that the expression level of *Gus* in the half of leaves under light was significantly higher than the half that was not under light (Supplementary Figure S3). In other words, the light treatment significantly increased the promoter activity of *FaMYB10L*. Overall, these results indicate that *FaMYB10L* is a light-responsive gene that has an expression that correlates with light-induced anthocyanin accumulation in the petioles of cultivated strawberries.

### 2.4. Overexpression of FaMYB10L in Strawberry Fruits

**Figure 4:**
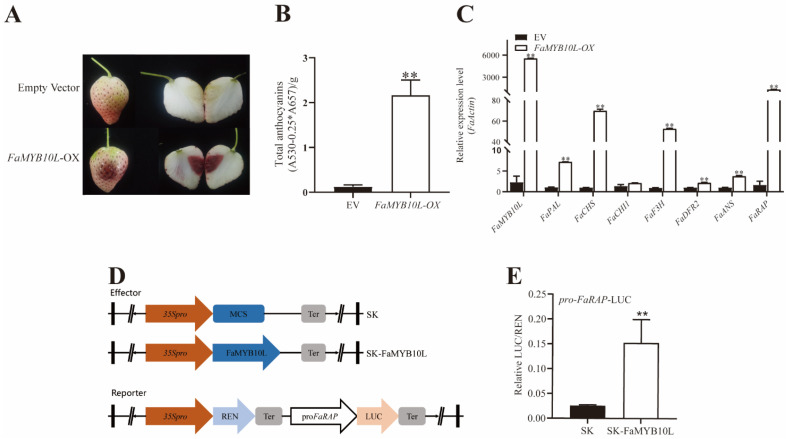
Effects of overexpression of FaMYB10L in strawberry fruits. (**A**) Phenotype of “Snow White” strawberries after injecting Agrobacterium containing pCAMBIA1301–FaMYB10L (*FaMYB10L*–OX) and empty vector. (**B**) Anthocyanin content of injection sites. (**C**) Expression levels of *FaMYB10L*, anthocyanin biosynthesis-related genes, and transporter genes in injection sites. *FaPAL*, phenylalanine ammonialyase; *FaCHS*, Chalcone synthase; *FaCHI*, chalcone isomerase; *FaF3H*, flavonol 3–hydroxylase; *FaDFR*, dihydroflavonol–4–reductase; *FaANS*, anthocyanidin synthase. (**D**) Schematic diagram of vectors for the dual luciferase assay. (**E**) Dual luciferase assay showed the FaMYB10L-activated promoter of *FaRAP*. Error bars for dual luciferase assays display the standard deviation (SD) from three separate experiments, each of which had six replicates. Error bars for expression level represent the SD of three independent replicates. ** *p* < 0.01 (two-tailed Student’s *t*-test).

We transiently overexpressed FaMYB10L in “Snow White” strawberry receptacles, which are a white receptacle strawberry variety. After 7 days, transient overexpression induced anthocyanin accumulation around the injection site, while no red colouration was observed around controls (Figure 4A). Further qRT–PCR analysis revealed that the expression of *FaMYB10L*, anthocyanin biosynthesis-related genes, and transport genes was upregulated. Anthocyanin accumulation also increased correspondingly (Figure 4B,C).

We chose *FaRAP* (anthocyanin transport gene) to further investigate the transcriptional activation mechanism (Figure 4D). The dual luciferase assays suggested that FaMYB10L activated the promoter of *FaRAP* (Figure 4E). In conclusion, FaMYB10L seems to promote anthocyanin accumulation by directly activating the promoter of anthocyanin biosynthesis-related genes and transport genes. 

### 2.5. Light Treatment of FaMYB10L-Overexpressing Calli

**Figure 5:**
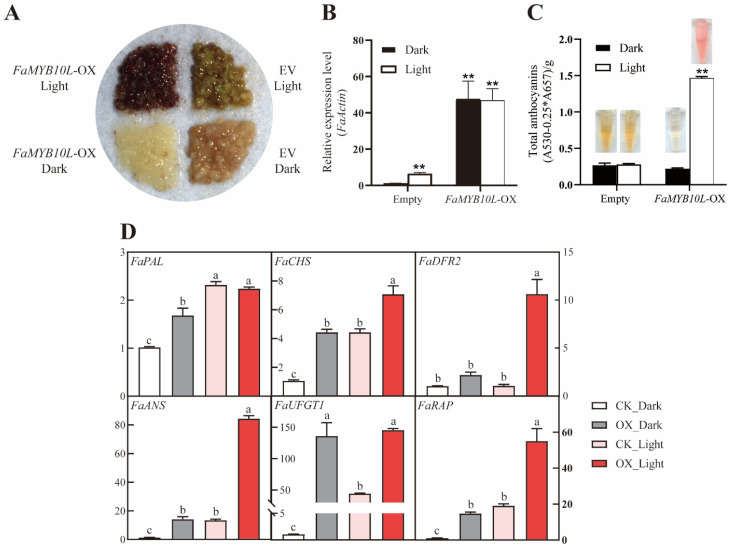
The effects of stable overexpression of FaMYB10L in strawberry calli under light treatment. (**A**) Anthocyanin accumulation in transgenic calli of *FaMYB10L*–OX and empty vector under light treatment; (**B**) Expression level of *FaMYB10L* in transgenic strawberry calli; (**C**) After exposure to light, the anthocyanin concentration of transgenic strawberry calli. Above each bar are the appropriate anthocyanin extracts. (**D**) Transcript levels of anthocyanin-related genes (*FaPAL*, *FaCHS*, *FaDFR2*, *FaANS*, *FaUFGT1*, and *FaRAP*) in strawberry calli. The error bars show three independent replicates. Different letters above the bars denote significantly different values (*p* < 0.05, Least Significant Difference (LSD) test). ** *p* < 0.01 (two-tailed Student’s *t*-test) compared with the empty vector control in the dark.

We transformed FaMYB10L into strawberries (“Benihoppe”) and obtained transgenic *FaMYB10L*-overexpressing calli lines (*FaMYB10L*–OX) (Figure 5A). Then, the calli were cultured for 7 days under continuous white light. *FaMYB10L* was shown to be highly expressed in transgenic calli under both dark and light conditions (Figure 5B). However, only under light conditions did anthocyanin significantly accumulate, while anthocyanin-related genes were highly expressed under these conditions (Figure 5C). Compared with the control (empty vector (EV)), anthocyanin accumulation in the *FaMYB10L*–OX calli was only obvious under light. There was a significant increase in the expression levels of anthocyanin biosynthesis-related genes *FaPAL*, *FaCHS*, *FaDFR2*, *FaANS*, and *FaUFGT1*, as well as the transport gene *FaRAP* in *FaMYB10L*–OX under light (Figure 5D). Together, our findings suggested that FaMYB10L was involved in light-induced anthocyanin accumulation in strawberries.

### 2.6. Transcriptome Analysis in FaMYB10L-Overexpressing Calli

**Figure 6:**
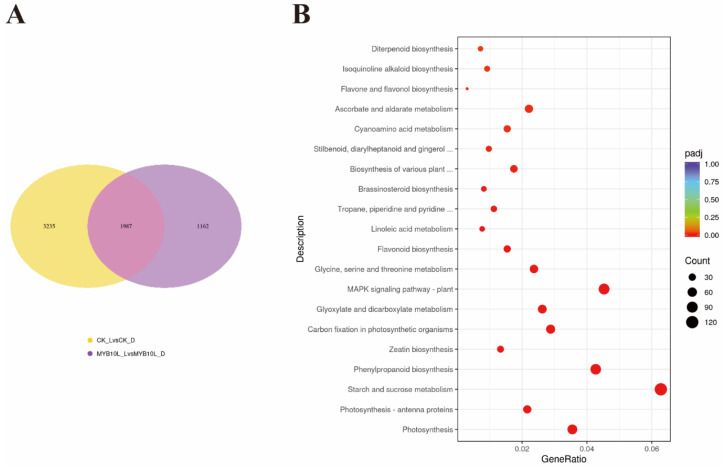
RNA–seq analysis of *FaMYB10L*–OX calli under light/dark conditions. (**A**) Venn diagram of the numbers of differentially expressed genes between empty vector and *FaMYB10L*–OX calli under dark/light conditions. (**B**) Enrichment analysis of KEGG pathways in the differentially expressed genes between empty vector and *FaMYB10L*–OX calli under light conditions.

Furthermore, the empty vector and *FaMYB10L*–OX calli after light treatment were subjected to RNA–Seq analysis (Supplementary Table S5). More than 5000 genes were affected by light treatment. Among them, 1987 genes overlapped with *FaMYB10L*–OX calli samples (Supplementary Table S1). A light-induced differential expression of 1162 genes has been identified specifically in *FaMYB10L*–OX calli (Figure 6A; Supplementary Table S1). The differentially expressed genes between the empty vector and *FaMYB10L*-OX calli during light treatment were also subjected to a KEGG enrichment analysis. With the exception of the flavonoid biosynthesis pathway, additional biological pathways, including starch and sucrose metabolism, the plant MAPK-signaling system, and phenylpropanoid biosynthesis all displayed comparatively high *p* values (Figure 6B; Supplementary Table S2). All of these findings provided additional evidence that FaMYB10L was a component of other pathways, in addition to flavonoid production.

### 2.7. FaMYB10L Interacted with bHLH3

**Figure 7:**
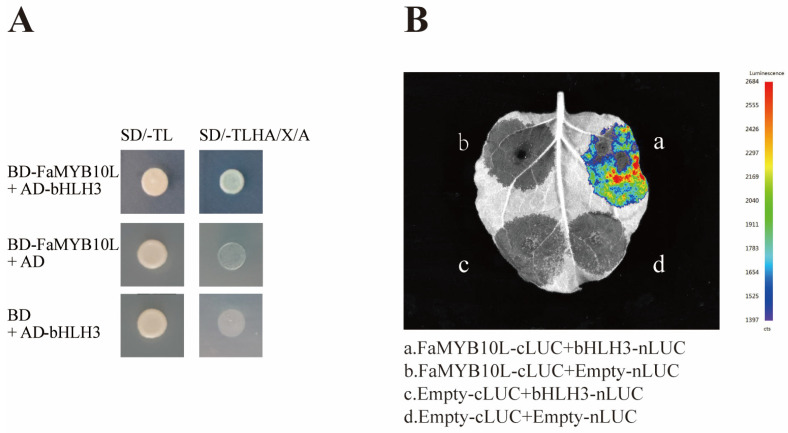
FaMYB10L interacts with bHLH3. (**A**) Yeast two-hybrid assays of FaMYB10L and bHLH3. SD/–TL: SD/–Trp-Leu, SD/–TLHA/X/A: SD/–Trp–Leu–His–Ade/X–α–Gal/AbA. (**B**) Firefly luciferase complementation imaging assay of FaMYB10L and bHLH3.

BHLH3 has been confirmed to interact with MYB to participate in the formation of the MBW complex in several Rosaceae, thereby regulating the biosynthesis of anthocyanin [19,34,35]. We investigated whether bHLH3 (Genebank, XM_004290615) can interact with FaMYB10L proteins. FaMYB10L interacted with bHLH3 in the yeast cells (Figure 7A). A firefly luciferase complementation imaging assay further confirmed their interaction (Figure 7B).

## 3. Discussion

Anthocyanins determine the appearance and quality of strawberry fruit. Anthocyanins also play an important function in different tissues [36,37]. In recent years, the anthocyanin accumulation of strawberry reproductive tissues has attracted more attention, while, vegetative tissues are also rich in anthocyanin. The accumulation of anthocyanin could protect plants from strong light stress [38,39].

R2R3–MYB is the most important transcription factor in anthocyanin biosynthesis regulation of four MYB subfamilies [40]. Whether in model plants or horticultural crops, many anthocyanin-related R2R3–MYB TFs have been isolated and identified [41,42,43,44]. Since the identification of ZmC1, many R2R3–MYB TFs have been characterized in the plantae [45]. For example, SlAN2 is an important part of the anthocyanin biosynthetic transcriptional network in tomatoes [46], while AtPAP1 is a critical resource for research on anthocyanin [47]. We found that FaMYB10L had a complete R2R3–MYB domain, while phylogenetic analysis showed that it was homologous to genes related to anthocyanin accumulation (Figure 1), so FaMYB10L was identified as a candidate gene for regulating anthocyanin accumulation. FaMYB10L shared 89.4% homology with FveMYB10-like, suggesting FaMYB10L may play a similar role in petioles of cultivated strawberries [32]. In addition, FaMYB10L shared just 44.40% amino acid sequence similarity with FaMYB10, indicating that *FaMYB10L* is a novel MYB gene independent of *FaMYB10*. An abnormal premature termination of FaMYB10 resulted in a white octoploid strawberry variety “Snow White” [24]. In this *myb10* mutant, FaMYB10L could rescue anthocyanin accumulation, indicating they are paralogous with similar functions on anthocyanin accumulation. Meanwhile, qPCR indicated FaMYB10L could upregulate transcript levels of anthocyanin biosynthesis-related genes: *FaPAL*, *FaCHS*, *FaDFR2*, *FaANS*, and *FaUFGT1*. In addition, GST proteins perform crucial functions in the transportation and storage of anthocyanins. FaMYB10L directly targeted the promoter of the anthocyanin transport gene *FaRAP*, according to a dual luciferase assay (Figure 4). The above results indicated that FaMYB10L was a positive regulator of anthocyanin accumulation by activating anthocyanin biosynthesis-related genes and transport genes independently of FaMYB10. Moreover, He et al. [48] constructed a new gene *RUBY* that produced a red bright red betalain, which was used as a very effective marker for selecting transformation in both rice and Arabidopsis. Here, anthocyanin accumulation was evident in stable overexpression strawberry calli (Figure 5A). FaMYB10L has the potential to be used to visualize gene expression without the use of chemicals or specialized tools as a visible marker for transgenes.

Using high-quality genomic analysis, it was found that MYB gene clusters, which are usually highly homologous, are arranged next to each other in tandem on the genome [49]. In many studies, they controlled tissue-specific accumulation of anthocyanin. *DcMYB7* and *DcMYB11c* are arranged in the P3 region of the carrot genome, which is a cluster of six MYB TFs that exists in ~0.5 Mb region control anthocyanin biosynthesis. The DcMYB7 controls anthocyanin accumulation in the taproots of purple carrots. When *DcMYB7* was knocked out, the petioles still appeared purple, while DcMYB11c is confirmed to function [50]. *Ruby1* and *Ruby2* are both located on Chr6 in Citrus. *AbRuby2^Full^* encodes an anthocyanin activator that functions in the pigmented leaves of Chinese box oranges, whereas *AbRuby1* is involved in the fruit pigment. [51]. In both of the recently reported diploid wild strawberry studies [32], as well as our study, *MYB10L* resides near *MYB10* on Chromosome 1, which is paralogous (Figure 1C). MYB10 is the dominant gene function in fruits, while MYB10-like is dominant in petioles. In summary, these gene duplication events, which regulate tissue-specific anthocyanin accumulation individually, frequently occur in the evolutionary development of anthocyanin-activating MYB genes. 

Light is an important environmental factor affecting anthocyanin biosynthesis, while plants have evolved a complete and conserved light response system. With the in-depth study on the cooperation of environmental factors and transcription factors, light-induced anthocyanin accumulation has been found to involve a large number of MYB TFs [52,53,54]. In the “Red Zaosu” pear fruit peels, PpMYB17 was associated with flavonoid accumulation and was significantly increased in response to light [55]. In eggplant, light-responsive SmMYB35 stimulates anthocyanin accumulation by activating *SmCHS*, *SmF3H*, *SmDFR*, and *SmANS* promoters [56]. *MdMYB1* transcript levels increased in apple fruit exposed to sunlight, increasing anthocyanin accumulation [17]. Here, the expression of *FaMYB10L* was induced when strawberry petioles were exposed to light (Figure 3); transiently *ProFaMYB10L*::*Gus*-expressing tobacco leaves showed higher *Gus* expression levels under light (Supplementary Figure S3), while the promoter analysis of *FaMYB10L* revealed a number of elements that respond to light. (Supplementary Table S4). In addition, light enhanced the anthocyanin accumulation of *FaMYB10L*–OX strawberry calli (Figure 5). These results showed that FaMYB10L is a component of light signaling.

In many plants, bHLH has been shown to interact with key MYB transcription factors to form MBW complex to regulate secondary metabolism [57,58,59,60]. MdMYB305 competes with MdMYB10 to bind MdbHLH33, thereby balancing the accumulation of sugars and anthocyanins in the fruit [61]. More previous studies on MYB–bHLH focused on anthocyanin biosynthesis. MdMYB10 could not efficiently stimulate anthocyanin biosynthesis in transient assays without the co-expression with MdbHLH3 and MdbHLH33 [19]. In addition, MdMYB9/11 and FaMYB5/9/10/11 also have been demonstrated to interact with bHLH3 to increase anthocyanin accumulation [62,63]. In our study, FaMYB10L showed no transacting ability in yeast cells (Figure 2A), suggesting that FaMYB10L may need to interact with other proteins to function. The conserved MYB sequence plays a role in the interaction between MYB and bHLH proteins: [DE]LX(2)[RK]X(3)LX(6)LX(3)R, and FaMYB10L has the conserved motif. A Y2H assay and the LCA assay suggested that FaMYB10L could interact with bHLH3 (Figure 7). These results suggested that the regulatory model of MYB participating in the regulation of anthocyanin biosynthesis is highly conserved among different species.

## 4. Materials and Methods

### 4.1. Plant Materials and Light Treatment

Cultivated strawberry (*Fragaria × ananassa*) varieties “Snow White”, “Benihoppe”, and tobacco (*Nicotiana benthamiana*) plants were grown at Sichuan Agricultural University greenhouses. Organs (petioles, runners, leaves, and flowers) and different developmental stages of fruits of “Benihoppe”, big green (14 days after fruit setting, DFS), and full red (28 DFS) used to analyse tissue-specific gene expression were collected from “Benihoppe”.

Cultured “Benihoppe” seedlings with strong growth and similar states were selected. After balancing under darkness for 4 d, the plants were treated under continuous light (light intensity was 2500 lx). Temperature and humidity were maintained at 22 °C and 70%, respectively. Petioles were collected as samples to analyse the expression level of *FaMYB10L*. Transgenic calli containing empty vector and FaMYB10L were cultured in the dark for 40 days; dark treatment was continued for culture; light treatment was white for 16 h/dark for 8 h; and light intensity was 2500 lx. Temperature and humidity were maintained at 22 °C and 70%, respectively. After 7 days of treatment, samples were acquired and phenotypic changes were recorded.

### 4.2. Isolation and Sequence Analysis

Using a Plant Total RNA Isolation Kit Plus (Foregene, Chengdu, China), total RNA was extracted from strawberry tissues. NanoDrop™ One microvolume UV–Vis spectrophotometer (Thermo Fisher Scientific, Waltham, MA, USA) was used to assess RNA integrity and concentration. Meanwhile, 1μg of RNA was used to synthesize cDNA using RT EasyTM II with the gDNase Reagent Kit (Foregene, Chengdu, China). The 10 × cDNA diluent was served as a template for gene amplification. The amplification products were then sequenced (Tsingke Biotech, Beijing, China) after being cloned and inserted into the Hieff Clone^®^ Topo-blunt simple vector (Yeasen, Shanghai, China). The primers used are listed in Supplementary Table S3. Multiple sequence alignments and construct phylogenetic tree using neighbor-joining and 1000 bootstrap replicates were conducted using MEGA 7.0 software.

### 4.3. Transcriptional Activity Analysis and Yeast Two-Hybrid Assay

The assays were conducted in accordance with the manufacturer’s guidelines (Takara, Beijing, China). The constructs pGBKT7–FaMYB10L were used for analysis. Yeast cells transformed with pGBKT7–FaBBX22 and pGBKT7–Lam were used as positive control and negative control, respectively. To verify the interaction of FaMYB10L and bHLH3, full-length bHLH3 was inserted into the pGADT7 vector. Then, pGBKT7–FaMYB10L and pGADT7–bHLH3 were cotransformed into the yeast strain Y2HGold. 

### 4.4. Strawberry Protoplast Transient Transformation and Subcellular Localization

The strawberry protoplasts were isolated from leaves of aseptic tissue culture seedlings and transiently transformed by a previously described method [64]. An EGFP fusion expression construct (driven by the CaMV 35S promoter), the pYTSL–16 vector, was modified to incorporate the full-length CDS of FaMYB10L using a homologous recombination method. PYTSL–16–FaMYB10L and a NLS–RFP (nuclear location marker) were transformed into strawberry protoplasts. A confocal laser-scanning microscope (FV3000, OLYMPUS, Tokyo, Japan) was used to visualize strawberry protoplasts after 18 h.

### 4.5. qRT–PCR Analysis

The 10 × cDNA diluent was served as a template for qRT–PCR. TB Green^®^ Premix Ex Taq™ (Takara, Beijing, China) was used to detect on a CFX connect real-time PCR detection system (Bio-rad, Hercules, CA, USA). The reaction system and procedure are conducted according to the instructions. The *Actin* gene (accession: AB116565.1) was used as the reference gene. The relative expression levels of the genes were calculated using the 2^−∆∆CT^ method.

### 4.6. Transient Transformation of Strawberry Receptacles

The full-length CDS of *FaMYB10L* was inserted into the modified pCAMBIA1301 vector that was driven by the CaMV 35S promoter. Transformation of strawberry fruits was performed in accordance with a prior report, with fewer alterations [65]. *Agrobacterium tumefaciens* GV3101 harbouring the pCAMBIA1301–35S–NOS and pCAMBIA1301–35S–FaMYB10L–NOS (pCAMBIA1301–FaMYB10L) constructs were transiently overexpressed in strawberry white fruit stage detachable receptacles. The fruits were injected with Agrobacterium (OD_600_ = 0.8) at approximately 300 μL per fruit in cocultivation buffer (1 × MS, 2% sucrose, 100 mM MES, 2 M MgCl_2_, pH 5.6). After transfection, the stalk was wrapped with absorbent cotton to prevent dehydration. After incubation at 25 °C for one week, all of the injection sites were collected. Each construct included 12 individual fruits and was independently replicated three times.

### 4.7. Total Anthocyanins Extraction and Determination

Measurements of anthocyanins were made in accordance with a previous report [1]. Fresh tissue weighing about 0.2 g was added to 2 mL of an extraction solution (trifluoroacetic acid: formic acid: H_2_O: methanol = 1:2:27:70) and stored at 40 °C. The absorbance was determined using a UV (UV–1800PC, MAPADA, Shanghai, China) at 530 and 657 nm after 6 h. The following formula was used to determine the total anthocyanin content: Q_Total anthocyanins_ = [A530 − (0.25 × A657)]/M, where M is the fresh weight of samples (g), A530 and A657 are the absorbances at the appropriate wavelengths, and Q_Total anthocyanins_ is the total anthocyanins content.

### 4.8. Dual Luciferase Assay

According to a prior report [66], the assay was conducted. The constructs pGreenII 62–SK–FaMYB10L and pGreenII 0800–LUC–proFaRAP were transformed into *A. tumefaciens* GV3101 (pSoup-p19) individually. The Dual Luciferase Reporter Gene Assay Kit (Yeasen, Shanghai, China) was used in accordance with the manufacturer’s instructions to perform an agrobacterium transient injection into the plant *N. benthamiana*. The LUC and REN enzyme activity were assessed using the Assay Kit at 72 h after infiltration using a Thermo Scientific^TM^ Varioskan^TM^ LUX multimode microplate reader (Thermo Fisher Scientific, Waltham, MA, USA). The LUC and REN activities were examined in three independent assays, each of which included a minimum of six biological replicates.

### 4.9. Firefly Luciferase Complementation Imaging Assay

The constructs pCAMBIA1300–nLUC–FaMYB10L and pCAMBIA1300–cLUC–bHLH3 were transformed into *A. tumefaciens* GV3101, which were then coinfiltrated into leaves of *N. benthamiana*. After 42 h, the same position as the agrobacterial infiltration site was injected with 0.5 mM of luciferin (Yeasen, Shanghai, China). Ten minutes later, the results were collected and visualized using a GelView 6000Plus Smart Gel Imaging System (BLT PHOTON TECHNOLOGY, Guangzhou, China).

### 4.10. Generation of Transgenic Strawberry Calli

The 40-day “Benihoppe” strawberry tissue culture seedlings were selected for the transformation. The leaves were cut into leaf discs of 4 mm × 4 mm and cultured on the pre-culture medium (1 × MS, 2% sucrose, 7% agar, 4 mg/L TDZ, 0.5 mg/L IBA, pH 5.6) for 3 days in the dark. The construct pCAMBIA1301–FaMYB10L or pCAMBIA1301 empty vector was transformed into Agrobacterium tumefaciens GV3101. The Agrobacterium (OD_600_ = 0.5) was resuspended by a buffer (MS, 20 g/L sucrose, 100 μM AS, pH 5.6), and leaf discs were soaked into it for 30 min. Then, they were cultured on co-culture medium (pre-culture medium with 100 μM AS added) for 3 days in the dark. Next, they were transferred to delayed medium (pre-culture medium with 250 mg/L carbenicillin and 200 mg/L timentin added) in the dark for 7 days. At last, the discs were cultured on selected medium (delayed medium with 5 mg/L hygromycin added) and subcultured every 40 days. The newly subcultured calli in the same bottle were combined into a biological replication for the light treatment, and at least three biological replicates were analysed. The newly subcultured calli in the same bottle were combined into a biological replication for the light treatment, and at least three biological replicates were analysed.

### 4.11. RNA–Seq Analysis

We used transgenic calli harboring the empty vector and pCAMBIA1301–FaMYB10L vector after light treatment for RNA–seq analysis. RNAprep Pure Plant Plus Kit (Polysaccharides & Polyphenolics-rich) (Tiangen bioteach, Beijing, China) was used to extract RNA and RNA Nano 6000 Assay Kit of the Bioanalyzer 2100 system (Agilent Technologies, CA, USA) was used to assess RNA integrity. Library preparation and sequencing were performed by Novogene (Beijing, China) using Illumina NovaSeq 6000 sequencing (BIOZERON, Shanghai, China) with a 150-bp pair-end strategy. Fastp software v0.23.4 (parameter: fastp -g -q 5 -u 50 -n 15 -l 150) was used to obtain clean reads. The clean reads were mapped to the *F. ananassa* genome with v1.0.a2 annotation using HISAT2 v2.0.5 with default parameters [67]. The fragments per kilobase of transcript sequence per million base pairs (FPKM) approach was used to determine each gene’s expression level. The differential expression analysis was conducted using the DESeq2 R package (1.20.0). Significantly, DE genes were those with an adjusted *p*-value <= 0.05 found by DESeq2. ClusterProfiler R package was used to implement Gene Ontology (GO) enrichment analysis of DE genes and test the statistical enrichment of DE genes in KEGG pathways.

### 4.12. Transient Transformation of Tobacco Leaves

The strawberry DNA was extracted using a plant DNA extraction kit (Foregene, Chengdu, China) following the instructions provided. Strawberry DNA was used as a template for the amplification of sequences containing the 1816 bp region upstream of the FaMYB10L transcription start site, employing specific primers (Supplementary Table S3). The 40-day *Nicotiana benthamiana* was selected for the tobacco transformation. The construct *ProFaMYB10L*::*Gus* (replace the CaMV 35S promoter in front of Gus of pCAMBIA1301 vector with *FaMYB10L* promoter (1816 bp)) was transformed into *Agrobacterium tumefaciens* GV3101. The Agrobacterium (OD_600_ = 0.5) was resuspended by a buffer (H_2_O, 250 μM AS, 100 mM MES, 2 M MgCl_2_, pH 5.6) and injected into tobacco. To protect the tobacco leaf from light, one half of it was wrapped with foil and the other was exposed. It was treated under dark conditions overnight, then treated under continuous light (light intensity was 2500 lx). Temperature and humidity were maintained at 22 °C, 70%, respectively. The leaves were collected as samples to analyse the expression level of *Gus*.

### 4.13. Statistical Analysis

The data are presented as the mean ± standard deviation (SD). Using the Student’s *t*–test (** *p* < 0.01) and one-way analysis of variance (ANOVA) in SPSS Statistics software (SPSS 23.0, IBM, Chicago, IL, USA), the statistical significance of the differences was examined. Graphs were created using GraphPad Prism 8.

## 5. Conclusions

In this study, we discovered a highly expressed R2R3–MYB TF and FaMYB10L in the petioles and runners of cultivated strawberries, with its expression being induced by light. FaMYB10L positively regulates the expression of genes involved in anthocyanin biosynthesis and transport, leading to increased anthocyanin accumulation in a light-dependent manner in cultivated strawberry. These findings offer novel insights into the transcriptional regulation of light-induced anthocyanin accumulation in cultivated strawberries.

## Data Availability

The transcriptome data from this study have been uploaded to NCBI Sequence Read Archive 544 database under BioProject ID: PRJNA993995.

## Supplementary Materials

The following supporting information can be downloaded at: https://www.mdpi.com/article/10.3390/ijms242316561/s1.
