# Supplementary material for: A Novel R2R3–MYB Transcription Factor FaMYB10-like Promotes Light-Induced Anthocyanin Accumulation in Cultivated Strawberry
Source: Int J Mol Sci. 2023 Nov 21;24(23):16561. doi: 10.3390/ijms242316561 (PMC10706590; doi:10.3390/ijms242316561)
Supplement: Supplementary file 1 [file ijms-24-16561-s001.zip › Supplementary_Material.pdf]

Supplementary Material

>FaMYB10L

ATGGAGGTGAGAAAAGGCTCATGGACCAAAGAGGAAGATCATCTTCTCAGGAACTACA
TTGAGAAACATGGTGAGGGAAGATGGCACAAGGTTCCTCTCCAAGCAGGCTTGAACAG
ATGCAGAAAGAGCTGCCGAATCAGGTGGTTGAATTATTTGAAGCCAAACATCAAGAGA
GGAGACTTTGAAGAGGATGAAGTCGATCTAATGATCAGGCTTCGTAAGCTTTTGGGAAA
CAGGTGGTCTTTGATTGCTGGACGGCTTCCCGGAAGAACATCAAACGATGTGAAAAACT
ATTGGAGTGCTCGACGAAGAAAAAACATAAACTTTGGCATCACGAATCATAATTATAAG
CCTCCAGAAGTAACAAAGACTACAGTAATAAGACCTCGACCACGAACCTTCACCAAAAG
TTTGCATTATTTGAATGCTAAAGTTACGACTTCAAATCCTATTGAATTAGGAAACAGTTC
ATCGTCAACATCACCACCTATAGAGAATGGAATTGATGACTGGAAAACTCTACTACATG
AAGATGTCCTGACAAACTTTTGGGTTGAGGATACAGCCTCAATGTCAAGTATAGGTGTC
AATTCTACTGAGCAAGGTTTTGAAATGGACCTGTGGCAGTTTTTACAAGAAGAAACAAG
GCAATAA

Supplementary Figure S1. The coding sequences of FaMYB10L. The sequence from translation start site (ATG) to stop codon for FaMYB10L.

>proFaMYB10L

ATAAATGTTGCTTGTTTTTAATATTGTATAGTTTCTTGGTGGAGTATTTATGGAAACCAA
GTACCACTTTTGCAAAGAATGGCTAAAAGAATACTTGGTTTGACCACAAGTTCATTCGA
ATGTGAAAGAAATTGGAGTGCTTTTGAGGGGGTAAGTACTTATTTTATTTAGTTATGCAC
TAATACAAACTAGCAATATAGTTTATCAATTAATTCTTTTTCCCTAATGTAGATACATAC
AAAGAAAATGAATAGACTAGATACAACAAGGTTGAACAATTTAGTGTATGTCCAATTCA
ATGCAAGGATTATGAACAAGAAAAGAAGAGAGAAAGAGAAGAATGTGGATGTACTACT
TGCTAGTGAAGCAATTATGGCACAAGAATGGATTGTTTATGGTGGTGATGAAGATGTTG
AGTCGGATCTTACTAGTGAAATGGTTGGAGATGATTTGGGAGTGGGTAGTGGCTTAGAG
CCTCGGAGAAGTTCTAGACTTCAAGAAATTAGAGAACTTCATGAGGATGATTTTGTATC
GGATGAAGAGGAAGAAGATGAAATAGACTTTGAGTTTGAGACCGACACAGAAGAAGTG
CAAGAGGAATATGGAGAAGAAGAATTTGAGGCATAGCCGCATAGGCTAGCAAGAATCT
AATAATTTGTTTCTTTCAAATTGTTTTTTTTTTTATAATTTCTAAGAACATTGTTGTTAAA
CATTTTGTCTATCTTTGAAGTATTTTTGCTATTTTAGAACATAATAATGTTTTTTATAAAT
TTTATTTATATAATTATTTTAATTAAATAAATAAAAAATATAAAAAATAAAAAACCGCCT
AGGAGCCCGATTAATCCTCTGGGCGCTACGCGCTGGCTGTCGGCCCGACTAGCGCCTAA
CGTTTTTTAGAACATTGATGACTTGGATAAGTTAAATGTAAAAAGAGTGGATTGTTAAT
ATTAATCGATGTTTATCTTAATTTTGAAATGATAATACATTAAGTTAGTTTTTTATTTAAC
AATATATACTGATTTTCGTACTTATCTAAATTTGTATTTTACTAAATTAATTTTATCAATT
ATATATTTTGTTTTGTTACAGTTATCAGTGCCTATTAACAAAAGAATACAAAAAATATAG
AACCGTATCATTTGCTCCGGTCAAATGGCACGCTTAATAAAGTATATATATCTACGTTTA
ATAAAGTGTATCTAAAAAGGGTAACCTTTGGCCATATTTCTAGTAGTGATTTGTCAGTGT
ATACTAAAACTATGAAAAATGGAATGGTCCAGGAAGAAGTTTGCAATCATTGTCCTTTT
TTCTTTTCTTTTTTTGCACTAATTTTTTCGACTATCACTATTAGTAGTGTTAACTGGAGGG

GTCTCACTGATCCGTCAACAGTGTTTACATTTAACAGTATGTTATTTACAACTTAATCTTT
TTTTTCGATTACTAATTACATCTTAATCTGCACATGCATAAGTTATGTCAAAAATAACCA
CCGGGGAAACGGTGGGGTGGAAAGCACTGACTTCTCTAACCAAGAGGTCTGGTGTTCGA
ATCCCCATTAAGGCCTTGGTGGCCAGCCGGCGGTGGCCCGTGCTAGGCAGGTGTGCCTG
GTGTGTGGTGTTCGCTTTCATGCCCCGTGGCGATGCAGGGATTAGTCCGGCTGTGCTGGG
ATACCCTGCATGGGTGTGTGTGTTACCTAATCTAACAAAGGCTAACCCCCAATCAAAAA
AAAAAAAAAGTTATGTCAAAAATAGAACTACGTACCACTCAAAACGTATATCTGTGGAC
AGCAGAACTACACGTAGGTATGTCCGTATGTGTTCAATTAAAATCATTTTTGATGGATGT
GAAGCCTCATTGAGTTGCCTTGTACTGTTTGGCCGGGGAGCATTGAGATTATTATATAT

Supplementary Figure S2. The promoter sequence of *FaMYB10L*

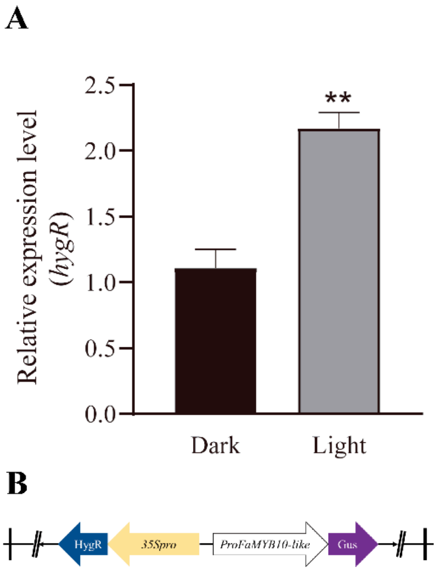

Supplementary Figure S3. (A)The expression level of *Gus*in transiently *ProFaMYB10L:: Gus*-expressing tobacco leaves under light/dark. *HygR* was the internal reference gene. (B) Schematic diagram of vectors. Error bars for expression level represent the SD of three independent replicates. ** $p < 0.01$ (two−tailed Student's t−test).

Data Availability Statement

The transcriptome data from this study have been uploaded to NCBI Sequence Read Archive 544 database under BioProject ID: PRJNA993995.
